# Association studies of the copy-number variable ß-defensin cluster on 8p23.1 in adenocarcinoma and chronic pancreatitis

**DOI:** 10.1186/1756-0500-5-629

**Published:** 2012-11-13

**Authors:** Stefan Taudien, Gabor Gäbel, Oliver Kuss, Marco Groth, Robert Grützmann, Klaus Huse, Alexander Kluttig, Andreas Wolf, Michael Nothnagel, Philip Rosenstiel, Karin Halina Greiser, Karl Werdan, Michael Krawczak, Christian Pilarsky, Matthias Platzer

**Affiliations:** 1 Genome Analysis, Leibniz Institute for Age Research - Fritz Lipmann Institute, Beutenbergstr 11, D-07745, Jena, Germany; 2 Department of Visceral, Thoracic and Vascular Surgery, University Hospital, Technical University of Dresden, Dresden, Germany; 3 Institute for Medical Epidemiology, Biostatistics and Informatics, Martin Luther University Halle-Wittenberg, Halle (Saale), Germany; 4 Institute for Medical Informatics and Statistics, Christian Albrechts University Kiel, Kiel, Germany; 5 Institute for Clinical Molecular Biology, Christian Albrechts University Kiel, Kiel, Germany; 6 German Cancer Research Centre, Division of Cancer Epidemiology, Heidelberg, Germany; 7 Department of Medicine III, Martin Luther University Halle-Wittenberg, Halle (Saale), Germany; 8 Cologne Center for Genomics, University of Cologne, Cologne, Germany

**Keywords:** Defensins, Single nucleotide variants, Copy number variation, Chronic pancreatitis, Pancreatic ductal adenocarcinoma

## Abstract

**Background:**

Human ß-defensins are a family of antimicrobial peptides located at the mucosal surface. Both sequence multi-site variations (MSV) and copy-number variants (CNV) of the defensin-encoding genes are associated with increased risk for various diseases, including cancer and inflammatory conditions such as psoriasis and acute pancreatitis. In a case–control study, we investigated the association between MSV in *DEFB104* as well as defensin gene (DEF) cluster copy number (CN), and pancreatic ductal adenocarcinoma (PDAC) and chronic pancreatitis (CP).

**Results:**

Two groups of PDAC (N=70) and CP (N=60) patients were compared to matched healthy control groups CARLA1 (N=232) and CARLA2 (N=160), respectively. Four *DEFB104* MSV were haplotyped by PCR, cloning and sequencing. DEF cluster CN was determined by multiplex ligation-dependent probe amplification.

Neither the PDAC nor the CP cohorts show significant differences in the *DEFB104* haplotype distribution compared to the respective control groups CARLA1 and CARLA2, respectively.

The diploid DEF cluster CN exhibit a significantly different distribution between PDAC and CARLA1 (Fisher’s exact test P=0.027), but not between CP and CARLA2 (P=0.867).

**Conclusion:**

Different DEF cluster b CN distribution between PDAC patients and healthy controls indicate a potential protective effect of higher CNs against the disease.

## Background

Pancreatitis, a necroinflammatory condition of the pancreas, has both acute and chronic manifestations. In the recent past, our understanding of the pathogenesis of pancreatic inflammation has improved considerably. Whereas acute pancreatitis is known to be initiated by premature activation of digestive enzymes within the exocrine component of the pancreas, chronic pancreatitis (CP) is characterized by progressive and irreversible damage to both the exocrine and endocrine components of the pancreas. CP is believed to result from repeated overt or silent episodes of acute pancreatitis [1]. The key histopathologic features of CP are pancreatic fibrosis, acinar atrophy, chronic inflammation, and distorted and blocked ducts [2]. The annual incidence of CP in industrialized countries ranges from 3.5 to 10 per 100,000. Alcohol abuse is the major risk factor for CP in Western countries, although other mechanisms such as mutations, pancreatic duct obstruction (caused by strictures), hypertriglyceridemia, hypercalcemia, and autoimmunity also have been implicated [3]. Since patients with CP have an approximately 13-fold higher risk to develop pancreatic cancer than the general population, the identification of disease-related genes is essential for understanding the transformation from benign to malignant disorder, and for developing strategies for early diagnosis [4]. Pancreatic cancer (pancreatic ductal adenocarcinoma, PDAC), is the fourth most common cause of cancer-related death in industrialized countries and characterized by extremely low survival rates [5,6]. Currently, no imaging procedure can reliably differentiate between benign and malignant tumors in CP patients.

Defensins are small cysteine-rich peptides that can be classified as either α-defensin or β-defensin, depending upon the arrangement of six critical cysteine residues. Defensins are synthesized as inactive preproproteins that become post-translationally activated. They are produced in the respiratory, gastrointestinal and genitourinary tracts, the skin, and by circulating blood cells. Defensins are considered a first line of defence against invading pathogens. Of all defensins, the ß-defensins comprise the largest group, with around 40 members encoded in the human genome. Most of the genes are located in defensin (DEF) clusters on chromosomes 8 and 20. In addition to their antimicrobial activity, β-defensins have multifaceted functions in innate and adaptive immunity [7]. The β-defensins are expressed in most epithelial cells and are found to be impaired in many inflammatory diseases, including Crohn's disease, psoriasis, pulmonary inflammation, and periodontal disease [8-13].

**Figure 1:**
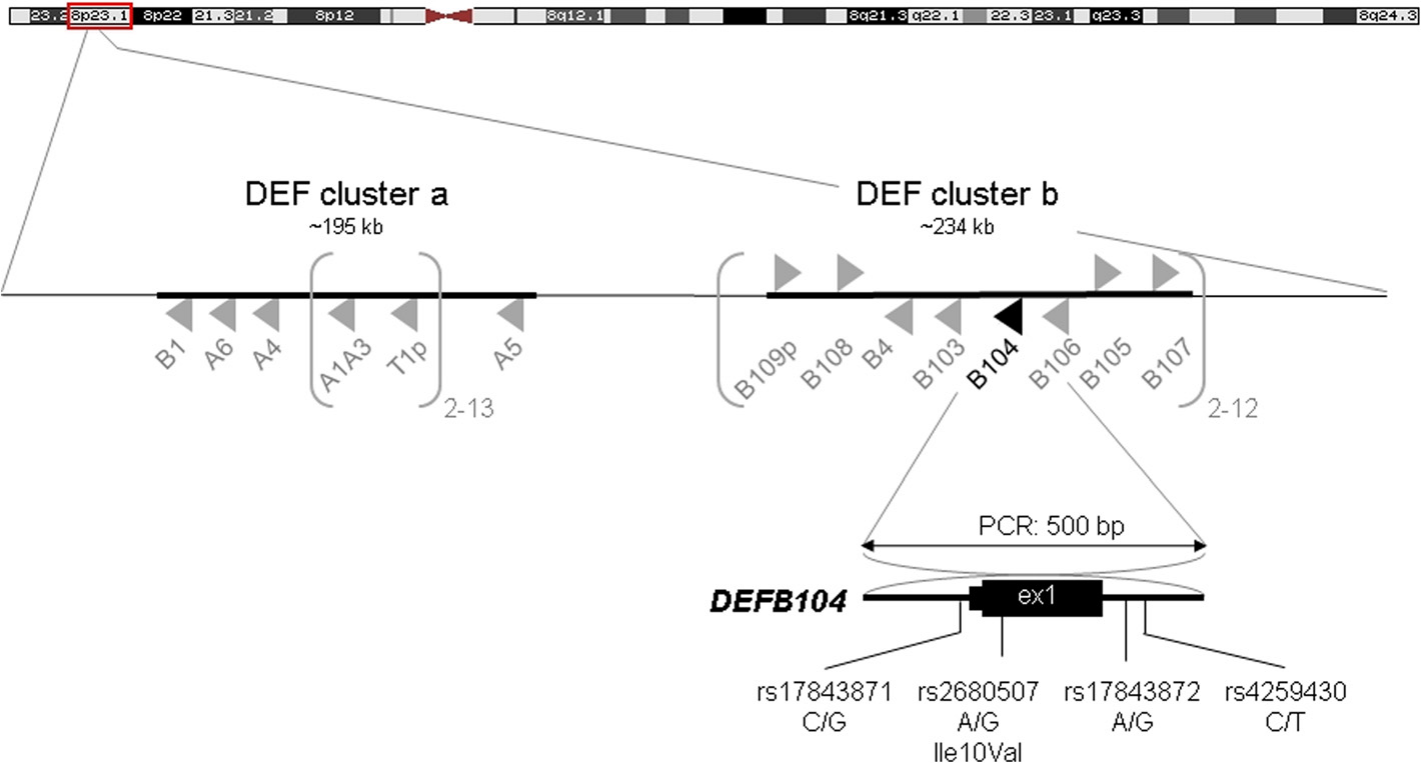
**Genomic organization of the human defensin (DEF) gene clusters on chromosome 8p23.1.** DEF cluster a is essentially single-copy, except a CNV region encompassing *DEFA1A3* and *DEFT1p* (2 to 13 copies per diploid genome), whereas DEF cluster b as a whole is CN-variable (2 to 12 copies). Black triangle (zoomed): *DEFB104* gene with the four multi site variations (MSV) investigated in this study. A: *DEFA*, α-defensin; B: *DEFB*, ß-defensin; ex = exon, p = pseudogene.

Except for *DEFB1*, all ß-defensin genes (*DEFB4*, *DEFB103109*) harbour a high degree of copy-number variation (CNV). Copy numbers (CN) range from 2 to 13 copies per diploid genome and show large inter-genic concordance because the respective genes bunch in a ~200 kb CNV region, called ‘DEF cluster b’ (Figure 1) [14-20]. In principle, both CNV and sequence variation within a given copy can contribute to clinical phenotypes through variation in gene expression [21]. So far, this has only been demonstrated experimentally for *DEFB4* but most probably applies to all other DEF genes with variable CN as well [17]. Anyhow, the analysis of genes located in CNV regions poses two methodological challenges: First, conventional genotyping of single nucleotide variations (SNV) cannot resolve whether a variation occurs between paralogs affected by CNV [22]. To acknowledge this problem, we will address SNVs within the copy number variable DEFB cluster as ‘multi-site variation’ (MSV). Second, the assessment of CNs is also complicated by methodological difficulties. Although widely used, qPCR has questionable reliability so that tightly controlled paralog ratio tests or multiplex ligation-dependent probe amplification (MLPA) have been recommended instead [23-28].

Disease association studies of the CN-variable ß-defensin genes are scarce. Hitherto reported associations mostly either lack replication or are conflicting [21,29-32]. No CN association, but a skewed distribution of MSV haplotypes, was identified in two prostate cancer groups [33]. The corresponding haplotypes comprise four MSV (rs17843871, rs2680507, rs17843872 and rs4259430) around exon 1 of *DEFB104*. While haplotypes GGGC and CAAT were significantly under-represented among patients, GAAT and GAAC were significantly over-represented. Moreover, high CNs of the ß-defensin cluster (≥9 copies per diploid genome) were found to be less frequent among prostate cancer patients than among healthy controls.

The aim of the present study was to search for associations between MSV-based *DEFB104* haplotypes and DEF cluster b CNs on the one hand, and pancreatic ductal adenocarcinoma and chronic pancreatitis on the other.

## Results

### DEFB104 haplotypes are not associated with PDAC or CP

Two independent cohorts of patients with pancreatic ductal adenocarcinoma (PDAC) and chronic pancreatitis (CP) were investigated in comparison with complementary age- and sex-matched healthy control groups [34] named CARLA1 and CARLA2, respectively (Table 1 and Methods section).

**Table 1:** Description of study groups

**Group**		**PDAC**	**CARLA1**	**CP**	**CARLA2**
Number (%)	male	37 (53%)	123 (53%)	49 (82%)	133 (83%)
	female	33 (47%)	109 (47%)	11 (18%)	27 (17%)
	**total**	**70**	**232**	**60**	**160**
Age (years)	minimum	41	46	36	45
	maximum	77	77	74	74
	mean	63.8	65.0	50.2	52.5
	median	67.0	66.8	49.0	50.0

PDAC: pancreatic ductal adenocarcinoma; CP: chronic pancreatitis; CARLA1, CARLA2: healthy control groups which were age- and sex-matched to PDAC and CP, respectively.

The haplotypes of four exon 1 MSV in *DEFB104* (Figure 1) were determined by PCR on the genomic DNAs from the four cohorts as well as from a commercially available pool of ~100 anonymous human DNAs. The PCR products were pooled by cohorts in equimolar amounts and cloned. Subsequently, clones were sequenced, haplotypes were inferred from the sequence traces and the haplotype fractions within cohorts were calculated (Table 2). Since these fractions do not take into account the effects of post-PCR pooling, however, they cannot be compared directly between patients and controls using standard statistical tests. Instead, the expected haplotype distribution under the null hypothesis had to be simulated as previously described [33]. An omnibus χ^2^ test based upon these simulations yielded a p value of 0.239 for the comparison of PDAC and CARLA1, and of 0.129 for CP and CARLA2, respectively, suggesting that there were no significant differences between the *DEFB104* haplotype distributions among cases and controls.

**Table 2:** **Haplotype frequencies (f_h_) as derived for *DEFB104 *MSV rs17843871, rs2680507, rs17843872 and rs4259430 by pooled PCR/cloning and Sanger sequencing**

**Haplotype**	**Frequency as determined by pooled PCR/sequencing**										**Group comparison**	
	**PDAC**		**CARLA1**		**CP**		**CARLA2**		**Human genomic pool**		**PDAC *vs *CARLA1**	**CP *vs *CARLA2**
	**No. clones**	**f**_**h**_	**No. clones**	**f**_**h**_	**No. clones**	**f**_**h**_	**No. clones**	**f**_**h**_	**No.clones**	**f**_**h**_	P*	P*
GAGC	224	0.40	286	0.34	310	0.41	391	0.34	413	0.38	0.160	0.061
GGGC	152	0.27	207	0.25	200	0.27	277	0.24	233	0.21	0.540	0.444
GAAT	39	0.07	92	0.11	92	0.12	153	0.13	106	0.10	0.109	0.747
CAGT	60	0.11	65	0.08	63	0.08	91	0.08	62	0.06	0.232	0.813
CAAT	29	0.05	48	0.06	27	0.04	76	0.07	47	0.04	0.773	0.123
CAGC	15	0.03	36	0.04	23	0.03	56	0.05	59	0.05	0.316	0.298
GAGT	22	0.04	28	0.03	9	0.01	41	0.04	54	0.05	0.721	0.079
GAAC	5	0.01	22	0.03	7	0.01	28	0.02	12	0.01	0.136	0.194
GGAT	3	0.01	22	0.03	2	<0.01	14	0.01	27	0.02	0.059	0.217
GGGT	7	0.01	10	0.01	2	<0.01	9	0.01	25	0.02	0.961	0.430
CGGC	6	0.01	5	0.01	2	<0.01	12	0.01	22	0.02	0.547	0.297
CGGT	0	0.00	5	0.01	1	<0.01	3	<0.01	0	0.00	0.163	0.720
GGAC	0	0.00	4	<0.01	0	0.00	0	0.00	6	0.01	0.222	n.a.
CAAC	1	<0.01	2	<0.01	0	0.00	2	<0.01	0	0.00	0.781	0.318
CGAT	1	<0.01	2	<0.01	0	0.00	0	0.00	4	<0.01	0.783	n.a
Total	564		834		738		1.153		1.070		0.239	0.129

* P values for the comparison of case and control haplotype frequencies were based upon simulations as previously described [33].

### DEF cluster b CN distribution differs between PDAC and controls

Diploid DEF cluster b CNs were determined by MLPA for 65, 232, 63 and 161 individuals from the PDAC, CARLA1, CP and CARLA2 groups, respectively (Additional files 1, 2, 3, 4 and 5). The median CN was 4 copies per diploid genome for all groups. CNs ranged from 2 to 7 in the PDAC group (mean: 4.22) and from 3 to 7 in the CP group (mean: 4.57). In both control groups, CNs were between 2 and 8 copies (mean CARLA1: 4.42, CARLA2: 4.55). Differences in mean CN between cases and control groups were not statistically significant (PDAC *vs* CARLA1: 0.20, P=0.151; CP *vs* CARLA2: 0.02, P=0.915) (Additional file 6).

**Figure 2:**
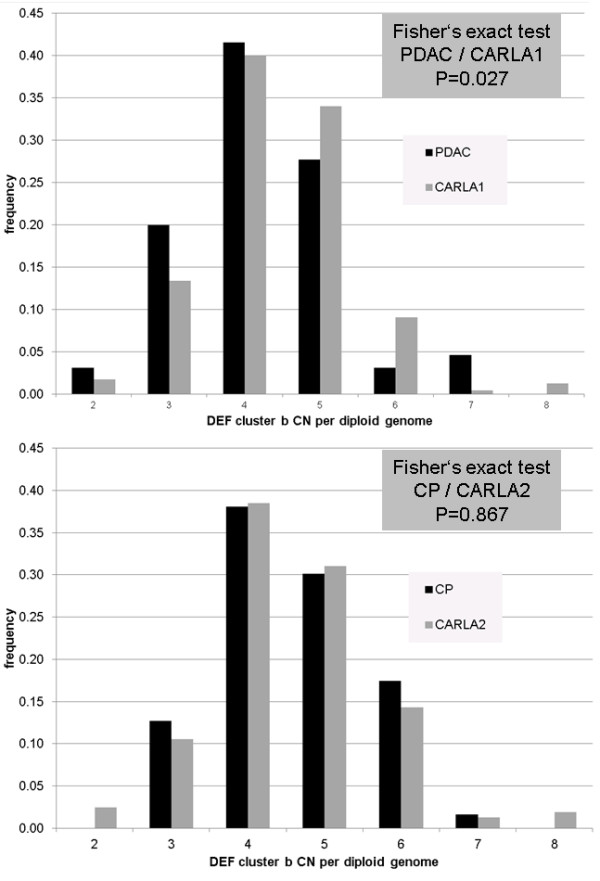
Distribution of DEF cluster b CN per diploid genome in the PDAC and CARLA1 (top) and CP and CARLA2 (bottom) cohorts.

The diploid CN distributions within the four cohorts are depicted in Figure 2. Application of Fisher’s exact test revealed that these distributions differed significantly between PDAC and CARLA1 (P=0.027), but not between CP and CARLA2 (P=0.867). The two control groups also did not differ significantly from each other (P=0.580).

## Discussion

Defensins are expressed in the pancreas although it is not entirely clear which cells actually produce and secrete which of these diverse peptides for which purpose. In pancreatic juice, only HNP-3 (α-defensin 3, encoded by *DEFA3*) has been detected but mRNA expression of ß-defensins has also been demonstrated in pancreatic tissue [35]. The important role of defensins in the innate immune system due to their antimicrobial, chemotactic and regulatory functions, and their involvement in inflammatory processes vindicates the assumption that defensins are also involved in the pathogenesis of pancreatitis and pancreatic cancer.

Pancreatitis may develop as a chronic disease after long-term alcohol abuse. Chronic pancreatitis is a strong risk factor for pancreatic cancer but alcohol does not appear to be an independent causative agent for the disease [36,37]. Interestingly, acute and chronic pancreatic inflammation occurs as an extra-intestinal co-morbidity of inflammatory bowel disease for which an involvement of defensins is also discussed [27,38,39]. Furthermore, in view of the microbicidal properties of defensins, it appears noteworthy that a link between infectious diseases and pancreatic cancer has been drawn both for viral diseases (mumps, HBV infections) and bacterial infections (*Helicobacter pylori*) [37,40].

In the present study, both sequence variants in a ß-defensin gene and CN variants of the cluster containing this gene were investigated for a putative association with PDAC and CP. As haplotyping of the CN-variable *DEFB104* gene was performed in pools, haplotypes cannot be assigned to the individual diploid CN for DEF cluster b. Respectively, both features had to be tested for association independently. All data from the patient groups were compared to age- and sex-matched healthy controls (CARLA1 and CARLA2).

Earlier, we have demonstrated association between *DEFB104* haplotypes and sporadic prostate cancer as well as under-representation of high diploid DEF cluster CN in patients with this disease [33]. In the present study, no statistical support was found for an association between *DEFB104* haplotypes and either PDAC or CP. However, analysis of the diploid CN distributions revealed a statistically significant difference between PDAC and CARLA1 that was due mainly to a paucity of 5- and 6-copy samples and an excess of 3-copy ones in the PDAC cohort.

Recently, under-representation of higher diploid *DEFB4* CNs (>4) was reported in patients with acute pancreatitis (AP) and severe acute pancreatitis (SAP) [41]. Since *DEFB4* is part of DEF cluster b and concordance for the CN of all genes within cluster b has been shown [16], this result is in agreement with our findings in the PDAC cohort. However, for CP, known to increase the risk for developing pancreatic cancer by 10 to 20-fold and a possible outcome of AP, we and others [42] did not observe significant associations with genetic features of the DEF cluster b, potentially pinpointing different roles of defensins in the etiopathogenesis of pancreatic diseases.

Although the functional consequences of the lower DEF cluster b CN observed for PDAC and AP are not yet resolved, lower CNs are rather associated with lower defensin expression [17]. In the light of inflammation as key feature of AP and the established link between inflammation and cancer [43], a low CN would be consistent with an anti-inflammatory effect of defensins described recently [44]. Assuming instead defensins to exert a pro-inflammatory effect [45] would favor a role of perturbed antimicrobial barrier defense in the etiopathogenesis of PDAC and AP. Further studies are necessary to find these missing functional links and to clarify which genetic variants may serve as reliable and feasible markers in the diagnosis and prognosis of pancreatic diseases.

## Conclusion

Different DEF cluster b CN distribution between PDAC patients and healthy controls indicate a potential protective effect of higher CNs against the disease. Replication of the study with larger sample numbers are needed to confirm the result and to draw definitive conclusions thereof.

## Methods

### Patients, DNA samples and Oligonucleotides

All individuals were of European origin. Cases of PDAC and CP were taken from two cohorts of patients with PDAC and CP who previously had undergone pylorus-preserving pancreatico-duodenectomy. They were complemented by age- and sex-matched healthy control groups sampled from the CARLA Study, a prospective cohort study of the general elderly population [34]. The sampled controls from CARLA were free from heart disease, cancer or any other severe chronic disease, and without intake of antiphlogistic medication (Anatomical Therapeutic Chemical Classification System (ATC) code A07). A description of the age- and sex-distribution of the groups is given in Table 1.

Genomic DNA was obtained from peripheral blood collected in EDTA tubes (QIAamp DNA Mini Kit). The studies were approved by the ethics committees of the Universities of Dresden (Vote No. EK96042007) and Halle (Vote No. 1983-01/07). Written informed consent was obtained from all participants. The funding sources of the study played no role in the study design, data collection, data analysis, data interpretation or writing of the report. A human genomic DNA pool derived from ~100 anonymous individuals (Roche Diagnostics, Cat.No. 1691112) served as an additional control. All primers were synthesized by Metabion AG (Martinsried, Germany).

### DEFB104 haplotyping

Amplification from individual genomic DNAs was carried out using primers 5'-TTCTGTAGCCCCAACACCTC-3' and 5'-GGTGCCAAGGACATCTAGGA-3', resulting in a 500 bp PCR product spanning four MSV (rs17843871, rs2680507, rs17843872, rs4259430) around exon 1 of *DEFB104* (GenBank Refseq NM_080389.2). PCR reactions were performed as described with the following cycling conditions: 95°C for 1 min; 5 cycles at 95°C for 30 s, 56°C for 30 s, and 72°C for 60 s, 27 cycles at 95°C for 30 s, 58°C for 30 s, and 72°C for 60 s, with a final extension at 72°C for 5 min [33]. The concentrations of PCR products were measured by use of a Nanodrop device and equal amounts were pooled per cohort. Pooled DNAs were cloned into pCR2.1-TOPO (Invitrogen) according to the manufacturer’s instructions and transformed into *E*.*coli* by electroporation. Well-isolated colonies were transferred and grown in LB broth supplemented with ampicillin. Plasmid DNA was isolated from the cultures by BioRobot 8000 and MagAttract 96 Miniprep Core Kit (Qiagen) and inserts were sequenced in both directions using M13 universal primers. Haplotypes were called by visual inspection of the sequence traces.

### DEF cluster copy numbers

For all individuals of the PDAC, CP, CARLA1 and CARLA2 cohorts, CNs of DEF cluster b (including *DEFB104*, Figure 1) were determined by multiplex ligation-dependent probe amplification (MLPA), using the P139 kit (MRC Holland), as previously described [16]. The MLPA probe set consists of 43 probes of which 10 are hybridizing to genes/pseudogenes within DEF cluster a, 10 to genes within DEF cluster b and 23 to *bona fide* single-copy genes flanking the defensin clusters as well as on other chromosomes, respectively. Peak areas were normalized against the summed peak areas of the “five nearest neighbor” (5nn) reference probes for each individual sample, relative locus doses were calculated and the diploid copy numbers were inferred. As internal quality control, four DNAs (NA18552, NA15324, NA12760, NA18858) with known CN (2, 4, 6 and 8, respectively) from commercially available lymphoblastoid cell lines (Coriell Cell repository http://www.coriell.org/) were used as copy number standards. Reliable copy number details from these samples are from independent, methodologically different determinations from different laboratories (see Table 2 in Groth et al. [2008] and references therein).

### Statistics

Following our PCR and sequencing approach, *DEFB104* haplotype determination is hampered by the fact that post-PCR pooling leads to an over-representation of alleles derived from individuals with low DEF cluster CN whilst alleles from genomes with high CN are under-represented. Furthermore, the number of sequenced clones differed considerably between the groups, ranging from 564 to 1153. This implies that haplotypes as called from sequence traces do not represent statistically independent observations and do not reflect the truly underlying haplotype distribution. Therefore, a haplotype-wise χ^2^ test could not be applied in the case–control comparisons. Instead, we simulated genotypes with respect to individual CN under the null hypothesis (i.e. no difference between cases and controls) and derived reference haplotype distributions for statistical testing from these simulated data, as previously described [33].

Differences in DEF cluster b CNs between cases and controls were first assessed by a comparison of the group-specific mean diploid CNs. Since the mean may not be a sufficient statistic for the underlying genotype distribution, diploid CN was also treated as a qualitative variable and gauged for statistically significant differences between groups using Fisher’s exact test as implemented in the SAS statistical analysis package V9.2 (SAS Inc., Cary, NY).

## Competing interests

The authors declare that they have no competing interests.

## Author’s contributions

GG, CP and RG collected the disease cohort’s samples and provided the patient’s data. KHG, KW, AK and OK conceived the CARLA study, did the cohort matching and provided the control samples. They also performed the statistical analyses with the support of MK, AW and MN. ST and MG carried out the molecular genetic studies with support by PR. ST, KH and MP drafted the manuscript. All authors read and approved the final manuscript.

## Supplementary Material

Additional file 1 Integer DEF cluster b copy numbers per diploid genome determined by MLPA, PDAC cohort. Click here for file

Additional file 2 Integer DEF cluster b copy numbers per diploid genome determined by MLPA, CP cohort. Click here for file

Additional file 3 Integer DEF cluster b copy numbers per diploid genome determined by MLPA, CARLA1 cohort. Click here for file

Additional file 4 Integer DEF cluster b copy numbers per diploid genome determined by MLPA, CARLA2 cohort. Click here for file

Additional file 5 Summary of DEF cluster b CN distribution. Click here for file

Additional file 6 Statistics for comparisons of DEF cluster b CN distribution. Click here for file
