# Supplementary material for: Association studies of the copy-number variable ß-defensin cluster on 8p23.1 in adenocarcinoma and chronic pancreatitis
Source: BMC Res Notes. 2012 Nov 13;5:629. doi: 10.1186/1756-0500-5-629 (PMC3532138; doi:10.1186/1756-0500-5-629)
Supplement: Additional file 1 — Integer DEF cluster b copy numbers per diploid genome determined by MLPA, PDAC cohort. [file 1756-0500-5-629-S1.pdf]

Additional file 1: Integer DEF cluster b copy numbers per diploid genome determined by MLPA, PDAC cohort

CN determination successfull: 65

	cohort	ID	CN (MLPA)
1	PDAC	2560	3
2	PDAC	2930	4
3	PDAC	3060	7
4	PDAC	3683	5
5	PDAC	3946	3
6	PDAC	4152	3
7	PDAC	4206	4
8	PDAC	4433	4
9	PDAC	4956	3
10	PDAC	7226	5
11	PDAC	7325	3
12	PDAC	7395	6
13	PDAC	7524	4
14	PDAC	7742	7
15	PDAC	7856	4
16	PDAC	7857	4
17	PDAC	8134	4
18	PDAC	8231	4
19	PDAC	8368	nd
20	PDAC	8857	nd
21	PDAC	8904	2
22	PDAC	9064	7
23	PDAC	9232	5
24	PDAC	9299	4
25	PDAC	9501	5
26	PDAC	9507	4
27	PDAC	9710	2
28	PDAC	9721	nd
29	PDAC	9734	4
30	PDAC	9938	4
31	PDAC	10050	5
32	PDAC	10067	4
33	PDAC	10403	3
34	PDAC	10448	4
35	PDAC	10655	5
36	PDAC	173559	4
37	PDAC	621849	nd
38	PDAC	627250	5
39	PDAC	628826	5
40	PDAC	631843	5
41	PDAC	631965	5
42	PDAC	635554	3
43	PDAC	639913	3
44	PDAC	643513	4
45	PDAC	645009	3
46	PDAC	646350	4

47	PDAC	646707	4
48	PDAC	649071	5
49	PDAC	650656	3
50	PDAC	656670	nd
51	PDAC	658988	4
52	PDAC	659343	5
53	PDAC	659667	5
54	PDAC	664953	5
55	PDAC	668064	3
56	PDAC	670441	nd
57	PDAC	679377	4
58	PDAC	683599	4
59	PDAC	683699	4
60	PDAC	687567	5
61	PDAC	690466	5
62	PDAC	694155	6
63	PDAC	T005	5
64	PDAC	T011	4
65	PDAC	T039	3
66	PDAC	T089	4
67	PDAC	T100	4
68	PDAC	T181	4
69	PDAC	T197	3
70	PDAC	T220	4
71	PDAC	T244	5
		average	4,21538462
		min	2
		max	7
		median	4,00
