# Supplementary material for: Association studies of the copy-number variable ß-defensin cluster on 8p23.1 in adenocarcinoma and chronic pancreatitis
Source: BMC Res Notes. 2012 Nov 13;5:629. doi: 10.1186/1756-0500-5-629 (PMC3532138; doi:10.1186/1756-0500-5-629)
Supplement: Additional file 2 — Integer DEF cluster b copy numbers per diploid genome determined by MLPA, CP cohort. [file 1756-0500-5-629-S2.pdf]

Additional file 2: Integer DEF cluster b copy numbers per diploid genome determined by MLPA, CP cohort

CN determination successfull: 63

	cohort	ID	CN (MLPA)
1	CP	3255	4
2	CP	3525	5
3	CP	3606	5
4	CP	3630	6
5	CP	3690	4
6	CP	3695	7
7	CP	3698	3
8	CP	3826	5
9	CP	3878	5
10	CP	3992	3
11	CP	4007	3
12	CP	4008	5
13	CP	4051	6
14	CP	4059	4
15	CP	4068	4
16	CP	4105	5
17	CP	4261	4
18	CP	4264	4
19	CP	4269	4
20	CP	4533	3
21	CP	4698	5
22	CP	4856	6
23	CP	4891	4
24	CP	4926	3
25	CP	4937	4
26	CP	4945	5
27	CP	4946	6
28	CP	5007	6
29	CP	5037	4
30	CP	5047	4
31	CP	5049	3
32	CP	5054	5
33	CP	7347	6
34	CP	7517	6
35	CP	8318	4
36	CP	9189	5
37	CP	9375	4
38	CP	9707	4
39	CP	10053	3
40	CP	10071	6
41	CP	10335	5
42	CP	10337	4
43	CP	199207	nd
44	CP	337456	5
45	CP	342023	4
46	CP	505226	5

47	CP	586129	5
48	CP	607752	4
49	CP	629988	6
50	CP	643449	5
51	CP	660891	nd
52	CP	664369	3
53	CP	686701	5
54	CP	725672	4
55	CP	T044	5
56	CP	T052	4
57	CP	T059	5
58	CP	T062	5
59	CP	T073	6
60	CP	T105	4
61	CP	T130	4
62	CP	T206	6
63	CP	T215	4
64	CP	T233	4
65	CP	T333	4
		average	4,57142857
		min	3
		max	7
		median	4,00
