# Supplementary material for: Association studies of the copy-number variable ß-defensin cluster on 8p23.1 in adenocarcinoma and chronic pancreatitis
Source: BMC Res Notes. 2012 Nov 13;5:629. doi: 10.1186/1756-0500-5-629 (PMC3532138; doi:10.1186/1756-0500-5-629)
Supplement: Additional file 3 — Integer DEF cluster b copy numbers per diploid genome determined by MLPA, CARLA1 cohort. [file 1756-0500-5-629-S3.pdf]

Additional file 3: Integer DEF cluster b copy numbers per diploid genome determined by MLPA, CARLA1 cohort

CN determination successfull: 232

	cohort	ID	CN (MLPA)
1	CARLA1	1100011	5
2	CARLA1	1100672	6
3	CARLA1	1102582	5
4	CARLA1	1105126	3
5	CARLA1	1108917	4
6	CARLA1	1110831	5
7	CARLA1	1122283	5
8	CARLA1	1122290	5
9	CARLA1	1122930	4
10	CARLA1	1200712	6
11	CARLA1	1201611	5
12	CARLA1	1202898	4
13	CARLA1	1204160	5
14	CARLA1	1206086	5
15	CARLA1	1207358	3
16	CARLA1	1208620	nd
17	CARLA1	1210538	4
18	CARLA1	1214358	5
19	CARLA1	2110247	5
20	CARLA1	2111519	3
21	CARLA1	2124255	4
22	CARLA1	2204184	6
23	CARLA1	2211184	4
24	CARLA1	2212440	5
25	CARLA1	2215012	2
26	CARLA1	2216276	4
27	CARLA1	2222012	4
28	CARLA1	2222650	4
29	CARLA1	2224577	4
30	CARLA1	2225855	nd
31	CARLA1	2226493	4
32	CARLA1	2228405	5
33	CARLA1	2230939	3
34	CARLA1	3100819	nd
35	CARLA1	3102014	5
36	CARLA1	3104562	6
37	CARLA1	3106489	6
38	CARLA1	3108318	4
39	CARLA1	3108382	5
40	CARLA1	3109000	4
41	CARLA1	3109022	4
42	CARLA1	3109648	5
43	CARLA1	3109660	4
44	CARLA1	3110278	4
45	CARLA1	3110290	4
46	CARLA1	3110930	5

47	CARLA1	3114112	4
48	CARLA1	3121721	5
49	CARLA1	3121744	6
50	CARLA1	3123654	5
51	CARLA1	3126799	5
52	CARLA1	3126865	6
53	CARLA1	3128143	4
54	CARLA1	3130045	3
55	CARLA1	3133782	5
56	CARLA1	3200857	4
57	CARLA1	3204217	4
58	CARLA1	3208698	5
59	CARLA1	3208706	4
60	CARLA1	3210614	4
61	CARLA1	3213140	3
62	CARLA1	3216321	5
63	CARLA1	3218231	4
64	CARLA1	3221411	4
65	CARLA1	3223918	4
66	CARLA1	4100260	2
67	CARLA1	4100276	3
68	CARLA1	4100900	5
69	CARLA1	4102039	4
70	CARLA1	4102068	5
71	CARLA1	4103932	4
72	CARLA1	4103961	6
73	CARLA1	4104593	4
74	CARLA1	4107150	7
75	CARLA1	4108409	2
76	CARLA1	4108993	4
77	CARLA1	4110949	6
78	CARLA1	4112180	5
79	CARLA1	4115349	4
80	CARLA1	4117319	4
81	CARLA1	4118595	3
82	CARLA1	4120505	5
83	CARLA1	4122421	4
84	CARLA1	4122993	4
85	CARLA1	4125610	5
86	CARLA1	4128754	4
87	CARLA1	4129386	5
88	CARLA1	4131928	5
89	CARLA1	4133206	4
90	CARLA1	4201741	6
91	CARLA1	4206193	5
92	CARLA1	4206833	3
93	CARLA1	4208105	4
94	CARLA1	4208714	4
95	CARLA1	4210020	5
96	CARLA1	4213170	5
97	CARLA1	4214471	4

98	CARLA1	4221442	4
99	CARLA1	4224653	3
100	CARLA1	4227150	4
101	CARLA1	4231618	4
102	CARLA1	4234203	5
103	CARLA1	5102099	4
104	CARLA1	5102722	5
105	CARLA1	5103354	3
106	CARLA1	5104610	4
107	CARLA1	5105270	nd
108	CARLA1	5105910	4
109	CARLA1	5107814	6
110	CARLA1	5108392	4
111	CARLA1	5109109	6
112	CARLA1	5112264	5
113	CARLA1	5112910	4
114	CARLA1	5114122	4
115	CARLA1	5114197	4
116	CARLA1	5114754	5
117	CARLA1	5114814	4
118	CARLA1	5116664	5
119	CARLA1	5116724	4
120	CARLA1	5119208	4
121	CARLA1	5119272	5
122	CARLA1	5121814	4
123	CARLA1	5122392	4
124	CARLA1	5124959	5
125	CARLA1	5126295	4
126	CARLA1	5126303	6
127	CARLA1	5126875	2
128	CARLA1	5127515	3
129	CARLA1	5128153	4
130	CARLA1	5130701	4
131	CARLA1	5201000	6
132	CARLA1	5201766	4
133	CARLA1	5203050	3
134	CARLA1	5203676	3
135	CARLA1	5205586	3
136	CARLA1	5206226	5
137	CARLA1	5206232	5
138	CARLA1	5206870	5
139	CARLA1	5207510	4
140	CARLA1	5208136	3
141	CARLA1	5210676	5
142	CARLA1	5211894	5
143	CARLA1	5212592	5
144	CARLA1	5213210	5
145	CARLA1	5214496	4
146	CARLA1	5214504	3
147	CARLA1	5215136	5
148	CARLA1	5215142	4

149	CARLA1	5215751	5
150	CARLA1	5217046	4
151	CARLA1	5217603	8
152	CARLA1	5217661	4
153	CARLA1	5218301	3
154	CARLA1	5218933	5
155	CARLA1	5219588	4
156	CARLA1	5221496	4
157	CARLA1	5222142	5
158	CARLA1	5222768	5
159	CARLA1	5222774	4
160	CARLA1	5223420	5
161	CARLA1	5223992	4
162	CARLA1	5224709	5
163	CARLA1	5225324	4
164	CARLA1	5225330	3
165	CARLA1	5225985	8
166	CARLA1	5227889	5
167	CARLA1	5228481	4
168	CARLA1	5229799	6
169	CARLA1	5230437	5
170	CARLA1	5231069	4
171	CARLA1	5231655	5
172	CARLA1	5231661	4
173	CARLA1	5232287	3
174	CARLA1	5233602	3
175	CARLA1	6100441	3
176	CARLA1	6101044	3
177	CARLA1	6101481	5
178	CARLA1	6104025	5
179	CARLA1	6105310	4
180	CARLA1	6105326	4
181	CARLA1	6107213	3
182	CARLA1	6107800	6
183	CARLA1	6107868	nd
184	CARLA1	6109761	4
185	CARLA1	6114160	4
186	CARLA1	6117387	6
187	CARLA1	6117967	5
188	CARLA1	6119883	5
189	CARLA1	6120573	3
190	CARLA1	6122490	4
191	CARLA1	6123130	5
192	CARLA1	6124996	6
193	CARLA1	6125056	5
194	CARLA1	6125694	5
195	CARLA1	6125702	4
196	CARLA1	6126280	5
197	CARLA1	6126340	5
198	CARLA1	6127606	4
199	CARLA1	6127612	5

200	CARLA1	6128899	4
201	CARLA1	6129462	4
202	CARLA1	6132079	8
203	CARLA1	6132642	4
204	CARLA1	6133334	6
205	CARLA1	6200480	5
206	CARLA1	6201113	5
207	CARLA1	6202443	4
208	CARLA1	6202450	5
209	CARLA1	6204991	3
210	CARLA1	6207541	4
211	CARLA1	6208813	4
212	CARLA1	6210030	4
213	CARLA1	6212000	5
214	CARLA1	6213263	5
215	CARLA1	6213903	5
216	CARLA1	6218929	6
217	CARLA1	6219633	4
218	CARLA1	6224090	5
219	CARLA1	6226656	4
220	CARLA1	6229206	5
221	CARLA1	6232958	4
222	CARLA1	7101218	4
223	CARLA1	7105400	5
224	CARLA1	7106032	4
225	CARLA1	7107209	4
226	CARLA1	7109220	3
227	CARLA1	7116740	6
228	CARLA1	7118006	5
229	CARLA1	7123119	3
230	CARLA1	7125680	5
231	CARLA1	7130770	3
232	CARLA1	7205716	5
233	CARLA1	7205722	4
234	CARLA1	7207626	5
235	CARLA1	7219606	3
236	CARLA1	7225995	3
237	CARLA1	7231725	4
		average	4,41810345
		min	2
		max	8
		median	4,00
