# Supplementary material for: Association studies of the copy-number variable ß-defensin cluster on 8p23.1 in adenocarcinoma and chronic pancreatitis
Source: BMC Res Notes. 2012 Nov 13;5:629. doi: 10.1186/1756-0500-5-629 (PMC3532138; doi:10.1186/1756-0500-5-629)
Supplement: Additional file 4 — Integer DEF cluster b copy numbers per diploid genome determined by MLPA, CARLA2 cohort. [file 1756-0500-5-629-S4.pdf]

Additional file 4: Integer DEF cluster b copy numbers per diploid genome determined by MLPA, CARLA2 cohort

CN determination successfull: 161

	cohort	ID	CN (MLPA)
1	CARLA2	1100028	4
2	CARLA2	1100040	5
3	CARLA2	1101281	4
4	CARLA2	1101938	4
5	CARLA2	1102560	3
6	CARLA2	1102576	3
7	CARLA2	1103185	6
8	CARLA2	1103200	4
9	CARLA2	1103819	4
10	CARLA2	1103825	4
11	CARLA2	1103831	5
12	CARLA2	1105741	4
13	CARLA2	1105758	2
14	CARLA2	1105764	5
15	CARLA2	1106380	8
16	CARLA2	1106404	4
17	CARLA2	1107651	4
18	CARLA2	1107668	5
19	CARLA2	1107674	5
20	CARLA2	1108283	4
21	CARLA2	1108290	5
22	CARLA2	1110191	5
23	CARLA2	1110200	6
24	CARLA2	1110216	4
25	CARLA2	1110854	4
26	CARLA2	1111492	5
27	CARLA2	1112103	3
28	CARLA2	1112110	5
29	CARLA2	1112126	5
30	CARLA2	1112735	6
31	CARLA2	1112758	5
32	CARLA2	1113396	4
33	CARLA2	1114036	6
34	CARLA2	1114668	4
35	CARLA2	1115283	4
36	CARLA2	1117187	4
37	CARLA2	1117827	5
38	CARLA2	1117833	5
39	CARLA2	1118471	4
40	CARLA2	1119105	4
41	CARLA2	1119111	5
42	CARLA2	1119750	5
43	CARLA2	1121013	4
44	CARLA2	1122308	4
45	CARLA2	1122923	5
46	CARLA2	1122946	5

47	CARLA2	1123584	2
48	CARLA2	1124218	6
49	CARLA2	1124862	4
50	CARLA2	1125494	nd
51	CARLA2	1126134	4
52	CARLA2	1126772	nd
53	CARLA2	1127412	6
54	CARLA2	1128699	4
55	CARLA2	1128707	8
56	CARLA2	1129322	4
57	CARLA2	1129954	5
58	CARLA2	1131230	6
59	CARLA2	1131879	4
60	CARLA2	1132494	5
61	CARLA2	1133140	5
62	CARLA2	1133766	4
63	CARLA2	1133772	5
64	CARLA2	1201605	4
65	CARLA2	1202243	4
66	CARLA2	1202875	5
67	CARLA2	1211800	4
68	CARLA2	1215607	5
69	CARLA2	1220063	3
70	CARLA2	1225184	4
71	CARLA2	1227734	2
72	CARLA2	1230914	4
73	CARLA2	2100102	3
74	CARLA2	2101350	6
75	CARLA2	2101969	3
76	CARLA2	2101975	4
77	CARLA2	2103879	4
78	CARLA2	2104519	4
79	CARLA2	2104525	5
80	CARLA2	2105134	6
81	CARLA2	2106435	5
82	CARLA2	2107044	4
83	CARLA2	2107050	4
84	CARLA2	2107647	4
85	CARLA2	2108345	5
86	CARLA2	2112140	4
87	CARLA2	2112157	3
88	CARLA2	2112772	6
89	CARLA2	2114647	4
90	CARLA2	2114707	4
91	CARLA2	2115902	4
92	CARLA2	2116540	4
93	CARLA2	2116617	5
94	CARLA2	2117858	4
95	CARLA2	2118504	5
96	CARLA2	2119739	4
97	CARLA2	2119797	6

98	CARLA2	2120429	5	
99	CARLA2	2121050	4	
100	CARLA2	2121707	7	
101	CARLA2	2122977	3	
102	CARLA2	2130592	5	
103	CARLA2	2133188	6	
104	CARLA2	2213102	4	
105	CARLA2	2225223	6	
106	CARLA2	2226501	3	
107	CARLA2	3100185	6	
108	CARLA2	3100200	4	
109	CARLA2	3102669	6	
110	CARLA2	3103290	6	
111	CARLA2	3103918	5	
112	CARLA2	3109594	5	
113	CARLA2	3114678	6	
114	CARLA2	3115301	4	
115	CARLA2	3117843	6	
116	CARLA2	3117910	5	
117	CARLA2	3118558	5	
118	CARLA2	3121106	5	
119	CARLA2	3126150	5	
120	CARLA2	3131955	4	
121	CARLA2	3132529	4	
122	CARLA2	3133167	4	
123	CARLA2	3205493	6	
124	CARLA2	3216315	5	
125	CARLA2	4213773	4	
126	CARLA2	1100011	5	*)
127	CARLA2	1100672	6	*)
128	CARLA2	1102582	5	*)
129	CARLA2	1105126	3	*)
130	CARLA2	1108917	4	*)
131	CARLA2	1110831	5	*)
132	CARLA2	1122283	5	*)
133	CARLA2	1122290	5	*)
134	CARLA2	1122930	4	*)
135	CARLA2	1200712	6	*)
136	CARLA2	1204160	5	*)
137	CARLA2	1206086	5	*)
138	CARLA2	1207358	3	*)
139	CARLA2	1208620	nd	*)
140	CARLA2	1210538	4	*)
141	CARLA2	1214358	5	*)
142	CARLA2	2110247	5	*)
143	CARLA2	2111519	3	*)
144	CARLA2	2124255	4	*)
145	CARLA2	3121744	6	*)
146	CARLA2	3128143	4	*)
147	CARLA2	3130045	3	*)
148	CARLA2	4100260	2	*)

149	CARLA2	4100282	nd	*)
150	CARLA2	4100900	5	*)
151	CARLA2	4102068	5	*)
152	CARLA2	4107150	7	*)
153	CARLA2	4117319	4	*)
154	CARLA2	4118595	3	*)
155	CARLA2	4128754	4	*)
156	CARLA2	4133206	4	*)
157	CARLA2	5201766	4	*)
158	CARLA2	5205586	3	*)
159	CARLA2	5219588	4	*)
160	CARLA2	5222142	5	*)
161	CARLA2	5225330	3	*)
162	CARLA2	5229799	6	*)
163	CARLA2	6101044	3	*)
164	CARLA2	6125694	5	*)
165	CARLA2	6132079	8	*)
		average	4,55279503	
		min	2	
		max	8	
		median	4,00	

*) these samples overlap with the CARLA1 control cohort
