# Supplementary material for: Association studies of the copy-number variable ß-defensin cluster on 8p23.1 in adenocarcinoma and chronic pancreatitis
Source: BMC Res Notes. 2012 Nov 13;5:629. doi: 10.1186/1756-0500-5-629 (PMC3532138; doi:10.1186/1756-0500-5-629)
Supplement: Additional file 5 — Summary of DEF cluster b CN distribution. [file 1756-0500-5-629-S5.pdf]

Additional file 5: Summary of DEF cluster b CN distribution

DEF cluster b CN	PDAC	f	CARLA1	f	CP	f	CARLA2	f
2	2	0,03	4	0,02		0,00	4	0,02
3	13	0,20	31	0,13	8	0,13	17	0,11
4	27	0,42	93	0,40	24	0,38	62	0,39
5	18	0,28	79	0,34	19	0,30	50	0,31
6	2	0,03	21	0,09	11	0,17	23	0,14
7	3	0,05	1	0,00	1	0,02	2	0,01
8			3	0,01			3	0,02
	65		232		63		161	
