# Supplementary material for: Association studies of the copy-number variable ß-defensin cluster on 8p23.1 in adenocarcinoma and chronic pancreatitis
Source: BMC Res Notes. 2012 Nov 13;5:629. doi: 10.1186/1756-0500-5-629 (PMC3532138; doi:10.1186/1756-0500-5-629)
Supplement: Additional file 6 — Statistics for comparisons of DEF cluster b CN distribution. [file 1756-0500-5-629-S6.pdf]

Additional file 6: Statistics for comparisons of DEF cluster b CN distribution

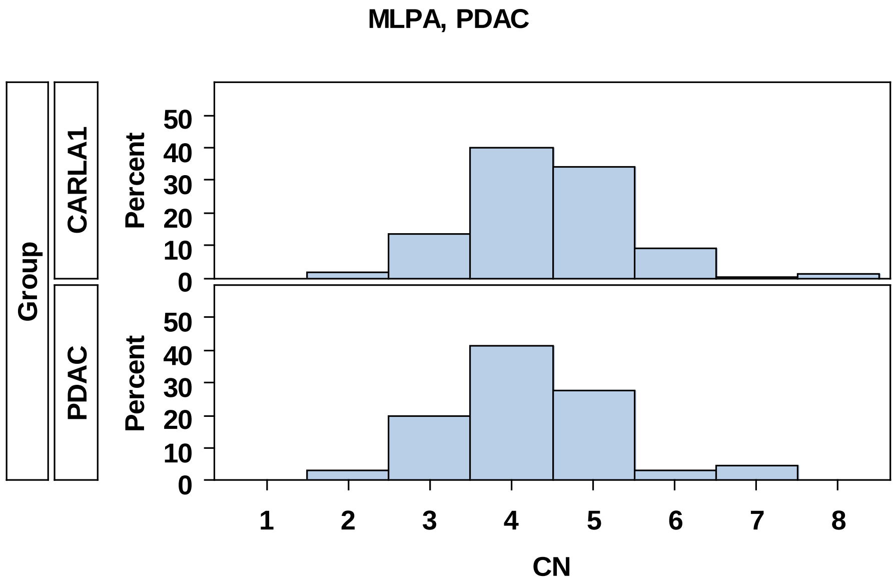

Mean difference in DEF cluster b CN (copies per diploid genome)

CARLA1- PDAC: 0.20

[95%-CI, p-value]: [-0.07; 0.48, p=0.15]

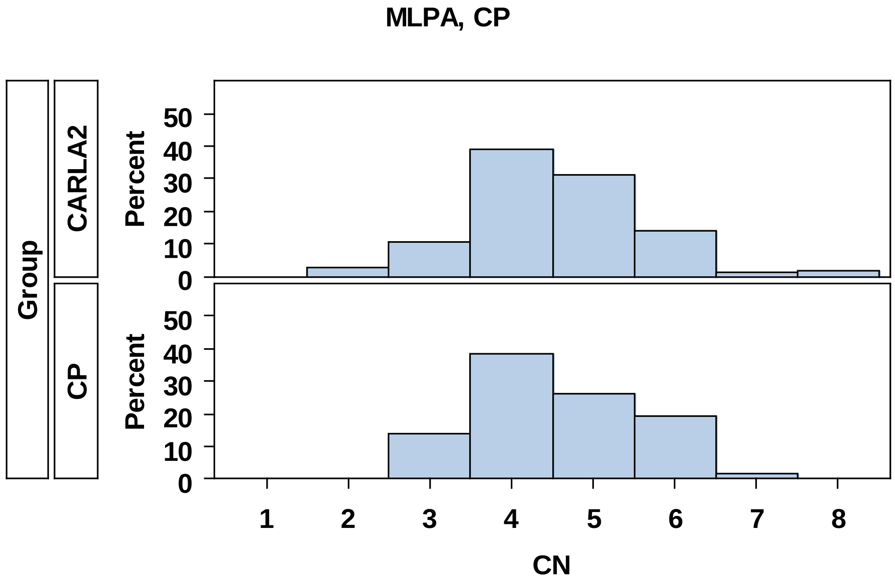

Mean difference in DEF cluster b CN (copies per diploid genome)

CARLA2- CP: -0.02

[95%-CI, p-value]: [-0.34; 0.31, p=0.92]
